# Missing data and chance variation in public reporting of cancer stage at diagnosis: Cross-sectional analysis of population-based data in England

**DOI:** 10.1016/j.canep.2017.11.005

**Published:** 2018-02

**Authors:** Matthew E. Barclay, Georgios Lyratzopoulos, David C. Greenberg, Gary A. Abel

**Affiliations:** a Cambridge Centre for Health Services Research, Department of Public Health and Primary Care, Forvie Site, Robinson Way, Cambridge, CB2 0SR, United Kingdom; b National Cancer Registration and Analysis Service, Public Health England, Victoria House, Capital Park, Fulbourn, Cambridge, CB21 5XA, United Kingdom; c Epidemiology of Cancer Healthcare and Outcomes (ECHO) Research Group, Department of Behavioural Science and Health, University College London, WC1E 7HB, United Kingdom; d University of Exeter Medical School (Primary Care), Smeall Building, St Luke’s Campus, Exeter, EX1 2LU, United Kingdom

## Abstract

**Background:**

The percentage of cancer patients diagnosed at an early stage is reported publicly for geographically-defined populations corresponding to healthcare commissioning organisations in England, and linked to pay-for-performance targets. Given that stage is incompletely recorded, we investigated the extent to which this indicator reflects underlying organisational differences rather than differences in stage completeness and chance variation.

**Methods:**

We used population-based data on patients diagnosed with one of ten cancer sites in 2013 (bladder, breast, colorectal, endometrial, lung, ovarian, prostate, renal, NHL, and melanoma). We assessed the degree of bias in CCG (Clinical Commissioning Group) indicators introduced by missing-is-late and complete-case specifications compared with an imputed ‘gold standard’. We estimated the Spearman-Brown (organisation-level) reliability of the complete-case specification. We assessed probable misclassification rates against current pay-for-performance targets.

**Results:**

Under the missing-is-late approach, bias in estimated CCG percentage of tumours diagnosed at an early stage ranged from −2 to −30 percentage points, while bias under the complete-case approach ranged from −2 to +7 percentage points. Using an annual reporting period, indicators based on the least biased complete-case approach would have poor reliability, misclassifying 27/209 (13%) CCGs against a pay-for-performance target in current use; only half (53%) of CCGs apparently exceeding the target would be correctly classified in terms of their underlying performance.

**Conclusions:**

Current public reporting schemes for cancer stage at diagnosis in England should use a complete-case specification (i.e. the number of staged cases forming the denominator) and be based on three-year reporting periods. Early stage indicators for the studied geographies should not be used in pay-for-performance schemes.

## Highlights

• Indicators of early stage at diagnosis are routinely reported for geographical populations.
• The current specification of these indicators results in biased and unreliable comparisons.
• Changes to the approach to handling missing data, and the reporting period are suggested.
• Organisational indicators for cancer care must address bias from missing data and low reliability.

## 1 Introduction

The percentage of cancer patients diagnosed at an ‘early stage’ (i.e. TNM stages 1–2) has been routinely reported for National Health Service commissioning organisations (Clinical Commissioning Groups, CCGs) since 2014 [1], following recommendations in the 2011 national cancer strategy for England [2]. Recently, this indicator has been adopted into a pay-for-performance scheme for CCGs [3]. Typical CCGs meeting the relevant targets in a given year would receive a financial incentive of £250,000. The aim of these public reporting and pay-for-performance schemes is to promote diagnosis of cancer at an earlier stage and thereby improve outcomes for patients across England. We further summarise this policy context and the technical aspects of the indicator in Box 1.

Box 1 **Early stage at diagnosis indicator**

In the English National Health Service (NHS), the planning, funding and monitoring of healthcare delivery is the responsibility of ‘healthcare commissioning’ organisations currently known as Clinical Commissioning Groups. These are responsible for geographically-defined populations. There are about 200 Clinical Commissioning Groups across England, covering an average general population of about 250,000 residents. To support and promote their planning, funding and monitoring function, high level performance indicators for Clinical Commissioning Groups are published annually, across different disease areas, including cancer. In England, a nationwide population-based cancer registration system has been in existence since 1971. In recent years, the modernisation of cancer registration systems has enabled the capturing of information on stage at diagnosis for a high proportion of patients. This has allowed for the introduction of the ‘early diagnosis’ indicator for Clinical Commissioning Groups studied in our paper. This indicator relates to the stage at diagnosis of 10 different solid tumour sites, and can be met by a Clinical Commissioning Group if either of the following criteria apply: a) 60% or greater proportion of all registered cases with relevant tumours are known to have been diagnosed in TNM stages 1 or 2; or b) there has been a 4% or greater absolute increase within a year in the proportion of all registered cases with relevant tumours known to have been diagnosed in TNM stages 1 or 2.

Alt-text: Box 1

Indicators used for comparing the performance of healthcare organisations should, among other considerations, be both valid and reliable. Valid indicators truly measure the intended construct of interest, while reliability indicates the precision by which the construct is measured. The validity of performance indicators based on routinely-collected healthcare data may be undermined by missing information [4], [5]. Low reliability, where measures are not precise enough to distinguish organisational performance, is a prevailing concern when person-level measures are aggregated into organisation-level scores [6], [7], [8], [9]. Frequently, indicators are published and used in pay-for-performance schemes without these concerns being examined or addressed.

The validity and reliability of the early stage indicator for CCGs as currently specified have not been evaluated. Currently, patients with cancer with no recorded stage are treated as though they had late stage cancer, but an alternate specification excluding such patients may be more appropriate. Furthermore, the annual reporting period may be either unnecessarily long or too short to allow for reliable estimation of performance. In this article, we demonstrate how appropriate statistical techniques may be used to examine the properties of this indicator, and identify specific improvements to reduce bias and improve its reliability.

## 2 Materials and methods

### 2.1 Data sources

We used population-based data (Public Health England National Cancer Registration and Analysis Service) on TNM stage at diagnosis and other patient and tumour characteristics of patients diagnosed during 2013 with 10 common cancers: bladder (ICD10 C67); female breast (C50); colorectal (C18–C20); endometrial (C54); lung (C33–C34); ovarian (C56–C574); prostate (C61); and renal (C64) cancers; melanoma (C43); and non-Hodgkin lymphoma (C82–C85). The choice of cancer sites and definition of early stage (TNM stages 1–2) reflected those included in the Public Health Outcomes Framework and the CCG Quality Premium; for both, data relating to patients diagnosed in 2013 was reported in 2014 [1], [3], [10], [11].

### 2.2 Analysis

#### 2.2.1 Examining bias arising from missing data in indicators of early stage at diagnosis

In the study year (2013) stage completeness across all 10 cancer sites was 82%, ranging from 71% to 91% for renal and endometrial cancer, respectively. We used multiple imputation by chained equations (MI) to produce a ‘best estimate’ early stage indicator, which we treated as the gold standard. Separately by cancer site, a binary early stage indicator for each patient was imputed with logistic regression [12], using auxiliary information on important patient and tumour characteristics associated with stage at diagnosis including patient age, sex, tumour grade (partially missing), CCG, and survival time from diagnosis [13], [14], [15], [16]. The MI indicator for each CCG was estimated as the mean percentage of tumours diagnosed at early stage over ten imputed datasets [17]. Appendix A contains further details of the imputation model.

We judged *a priori* that indicators based on the MI approach were not suitable for routine use in public reporting, primarily due to the need for follow-up periods to have elapsed to obtain survival information for use in imputation models, as well as the computational complexity and lack of end-user familiarity with the underlying statistical methods. Instead simpler approaches would be preferable if they are not associated with a substantial degree of bias. We therefore investigated the degree of bias in CCG scores using two simpler approaches for producing early stage indicators. First, the ‘missing-is-late’ indicator, where the percentage of all tumours with recorded early stage is estimated assuming that those without recorded stage information are advanced stage tumours. The missing-is-late approach is currently used to produce early stage indicators [1], [3], [10]. Second, the ‘complete-case’ indicator, where the percentage of staged tumours diagnosed at early stage is estimated based only on tumours with observed stage. We described the degree of bias in either missing-is-late or complete-case indicators by comparing organisational estimates against the ‘best estimate’ MI indicator.

#### 2.2.2 Examining the reliability of early stage indicators

The statistical reliability of a measure indicates its reproducibility (consistency) in repeated measurement and its robustness to random measurement error. Here we are concerned with organisation-level (or Spearman-Brown) reliability which represents the extent to which organisational measures (in our case the measured percentages of cancer patients diagnosed in early stage) reflect true differences between organisations, as opposed to random (i.e. chance) variation [7], [18], [19], [20]. For further details of the calculation of reliability for binary indicators, see Appendix B.

Mixed effects logistic regression models were used to model variation in the percentage of tumours diagnosed at early stage estimated using the complete-case indicator. Our main focus was the composite (all 10 cancers) indicator for CCGs, but we performed similar analyses for each individual cancer site (see Appendix B) and for local government organisations (local authorities) and general practices. These models produced an estimate of the organisation-level variance on the log-odds scale. The estimated variance was used to calculate odds ratios for diagnosis at early rather than late stage comparing the 75th/25th and 95th/5th percentiles of the distribution to illustrate the variation between organisations. Importantly, this was the underlying (true) variation which can be thought of as that which would be seen with very large sample sizes in each organisation, such that the influence of sampling variation would be minimal. This underlying (true) variation will be less than the variation in observed stage metrics as the latter will also include a contribution from chance/sampling [19]. The organisation-level variance on the log-odds scale was also used to calculate the reliability for each indicator based on the number of cases in the study year.

In addition to estimating the reliability of the observed data, model outputs were used to estimate the number of tumours required for each organisation to have a reliable estimate of the percentage diagnosed at an early stage based on reliability thresholds of 0.7 and 0.9. A reliability of 0.7 or higher is commonly required in public reporting, while a reliability of 0.9 may be required for high-stakes reporting, including pay-for-performance schemes [6], [19], [20], [21]. Following this we calculated the number of years of data required for reliable reporting at current completeness levels.

To illustrate the direct impact of low reliability, we used the estimated distribution of CCG performance in 2013 to evaluate expected misclassification rates for CCGs on the Quality Premium pay-for-performance thresholds. Estimating the overall CCG misclassification rate (in respect of both targets combined) was not possible using one year of data. We therefore performed two similar simulation processes, one for investigating the 60% criterion and one for the ≥4% change criterion (Appendix D). This proceeded as follows. We started with a list of 209 CCGs and the number of staged tumours (*N_i_*) in 2013 for each CCG. We simulated plausible values of the true performance of each CCG, *P_i_*, using the intercept and random effect from our multi-level model, and mapping back from the logistic to the probability scale. We used the binomial distribution with probability of success *P_i_* and number of trials *N_i_* to generate plausible observed performances for each CCG, given the simulated underlying performance and actual number of staged tumours. For the ≥4% change criterion we simulated two years of data for each CCG with a true, uniform change in performance between the two years, repeated for true changes between −4% and +12%, in steps of 0.1%. We repeated each simulation 10,000 times, examining the sensitivity, specificity, and positive and negative predictive values of both the 60% and ≥4% change criteria. All analyses were carried out in Stata 13 [22].

## 3 Results

**Fig. A1:**
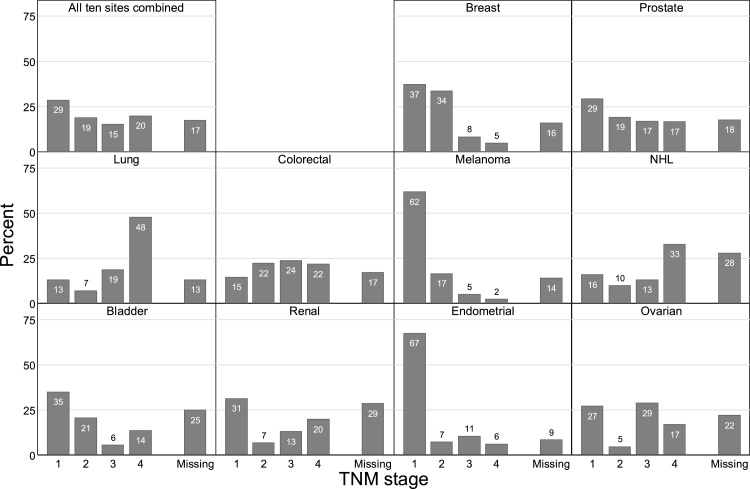
Percentage of tumours by stage at diagnosis, England 2013.

Of 208,112 diagnoses of relevant tumours in 2013, 98,218 (47%) were diagnosed in early stage (1–2), 71,809 (35%) were diagnosed in stages 3–4, and 38,085 (18%) had no recorded stage information (Fig. A1).

### 3.1 Bias arising from missing data in indicators of early stage at diagnosis

Comparing with the ‘best estimate’ indicator based on multiply imputed data for CCGs (median 55% early stage, range 45%–66%), the missing-is-late indicator underestimated true performance (median 48%, range 25%–62%), while the complete-case indicator overestimated true performance (median 57%, range 48%–70%).

**Fig. 1:**
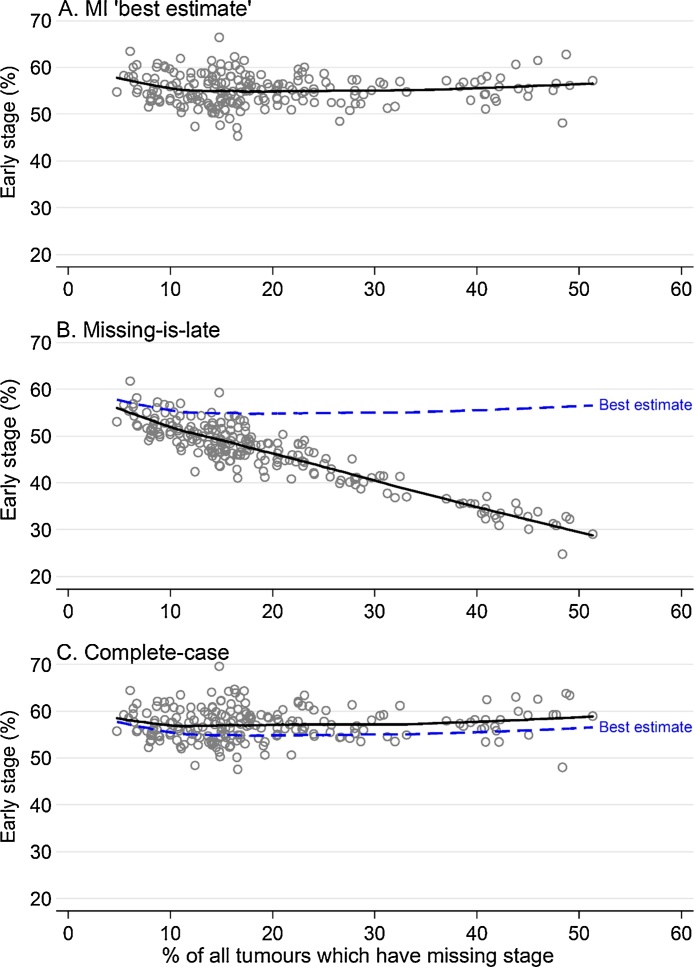
Observed early-stage percentage calculated using: A. the ‘best estimate’ multiple imputation approach; B. the missing-is-late approach; and C. the complete-case approach, plotted against the percentage of tumours with no recorded stage information, CCGs, England 2013.

There was little association between CCG early stage percentages estimated using the indicator based on multiply imputed data and CCG percentages of tumours with missing stage (Fig. 1 panel A). In contrast, when using the missing-is-late specification, we observed a very strong negative relationship between early stage and missing stage percentages (panel B). The complete-case specification did not show a clear association of these two measures (panel C).

**Fig. 2:**
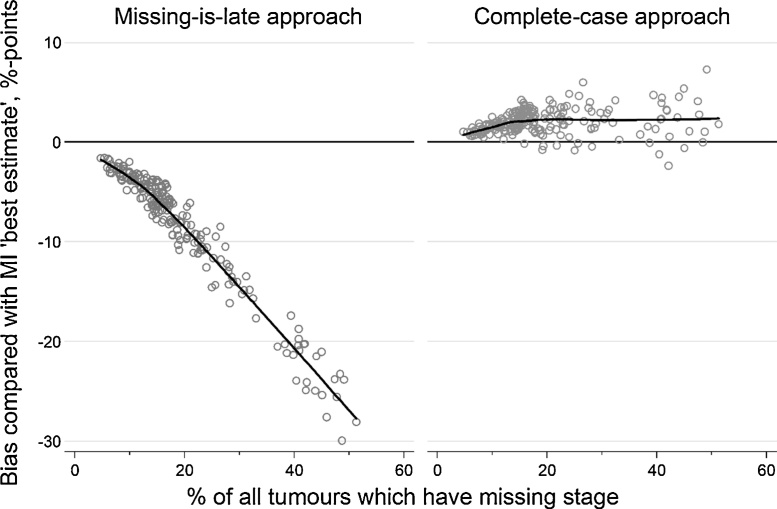
Bias in scores calculated using the complete-case and missing-is-late approaches when compared with the ‘best estimate’ MI indicator, plotted against the percentage of tumours with no recorded stage information, CCGs, England 2013.

Fig. 2 shows the bias associated with the amount of missing stage information compared with the ‘best estimate’ MI indicator (i.e. where bias is the difference between the ‘best estimate’ MI indicator and the indicator of interest). Bias in the missing-is-late specification increased in magnitude rapidly as the percentage of tumours with missing stage information increased; median bias across all CCGs was −6% (range −30% to −2%). Using a complete case specification typically produced less biased estimates than the missing-is-late approach across all CCGs, irrespectively of the degree of data completeness. There was a slight positive association between the degree of bias and the percentage of patients with missing stage among CCGs with <20% missing stage data, and no apparent association among CCGs with >20% missing stage data. Median bias in the complete-case specification across all CCGs was +2% (range −2% to +7%). Importantly, between-CCG variation in bias due to missing data under the missing-is-late specification (observed range of bias: 28%) was larger than observed variation in early stage on the ‘best estimate’ (observed range of performance: 21%), while this was not the case for the complete-case indicator (observed range of bias: 9%).

### 3.2 Reliability of the complete-case indicator

The median reliability of the early stage indicator for CCGs was 0.66 (Table 1), despite strong evidence of variation between CCGs (*p <* 0.0001) and moderate sample sizes for each CCG (median 691 staged tumours). This is below levels of reliability required for use in public reporting or pay-for-performance schemes. The aggregation of three years of data would suffice to produce indicators suitable for public reporting (*λ* ≥ 0.7) for 90% of CCGs. Indicators for 90% of CCGs with sufficient reliability for use in pay-for-performance schemes (*λ* ≥ 0.9) would require aggregation of nine years of data. Reliability estimates for individual sites are given in Table C1. For breast and lung cancer, indicators based on three and four years of incident cases respectively would allow for adequate reliability (*λ* ≥ 0.7) for about 70% of all CCGs, respectively. For other cancer sites, eight (renal cancer) to 35 (endometrial cancer) years would be required. Results for local authorities were similar, while general practice indicators had very low reliability (Table C2).

**Table 1:** Number of CCGs, staged tumours per CCG, odds ratios over estimated underlying distribution of CCG performance, quartiles of the reliability of the complete-case early stage indicator, and the number of tumours and associated aggregated years of data for 50%, 70%, 90% and 100% of CCGs to have reliability of 0.7 or higher or of 0.9 or higher.

CCGs		209
Number of staged tumours per CCG	Minimum	125
	25th percentile	479
	Median	691
	75th percentile	943
	Maximum	3575
Odds ratio over CCG distribution*	75th/25th percentiles	1.16
	95th/5th percentiles	1.43
Reliability	Minimum	0.26
	25th percentile	0.58
	Median	0.66
	75th percentile	0.73
	Maximum	0.91
Number of tumours per CCG required for reliability 0.7	50% of units	803
	70% of units	812
	90% of units	833
	All units	926
Data years required for reliability 0.7	50% of units	1.2
	70% of units	1.5
	90% of units	2.3
	All units	6.6
Number of tumours per CCG required for reliability 0.9	50% of units	3095
	70% of units	3132
	90% of units	3210
	All units	3570
Data years required for reliability 0.9	50% of units	4.5
	70% of units	5.6
	90% of units	8.7
	All units	25.3

* *p <* 0.0001. Odds ratio calculated directly from the estimated variance of the random intercept from the mixed-effects logistic regression ($\hat{\sigma^{2}}=$ 0.012) using the appropriate centiles of the standard normal distribution. The 75th/25th percentile odds ratio is calculated as $e^{\left(1.35\times \sqrt{0.012}\right)}$ and the 95th/5th percentile odds ratio is calculated as $e^{\left(3.29\times \sqrt{0.012}\right)}$.

### 3.3 Probable misclassification on CCG Quality Premium targets for reporting periods of varying length

**Fig. 3:**
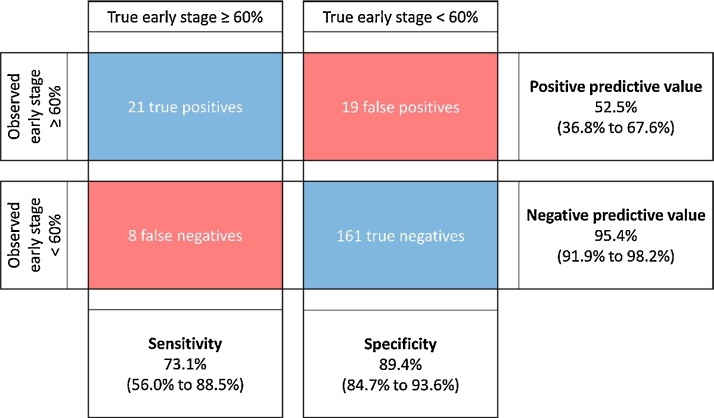
Estimated number of true positives, false positives, true negatives and false negatives, with associated sensitivity, specificity, positive and negative predictive values (95% confidence intervals), for the 60% early stage target given performance similar to 2013 and tumours counts as in 2013.

Considering the CCG Quality Premium criterion providing financial incentives to CCGs which have 60% of tumours diagnosed at stage 1 or 2 in a single year, based on our simulation (which assumes the complete-case indicator is used), we would expect 40 of the 209 CCGs to appear to meet this 60% target, of which only 21 would have an underlying or long-run performance of 60% or higher, giving a positive predictive value of 53% (Fig. 3). We would expect 29 CCGs to have underlying performance above the 60% target, of which one quarter (eight of 29) would appear to miss the target, giving a sensitivity of 74%. Aggregating multiple years of data reduces expected misclassification rates. Using 2.5 (9) years of data, giving reliability of 0.7 (0.9) for more than 90% of CCGs, increases the expected number of true positives to 23 (25) and reduces the expected number of false positives to 11 (5) (Table C3).

**Fig. 4:**
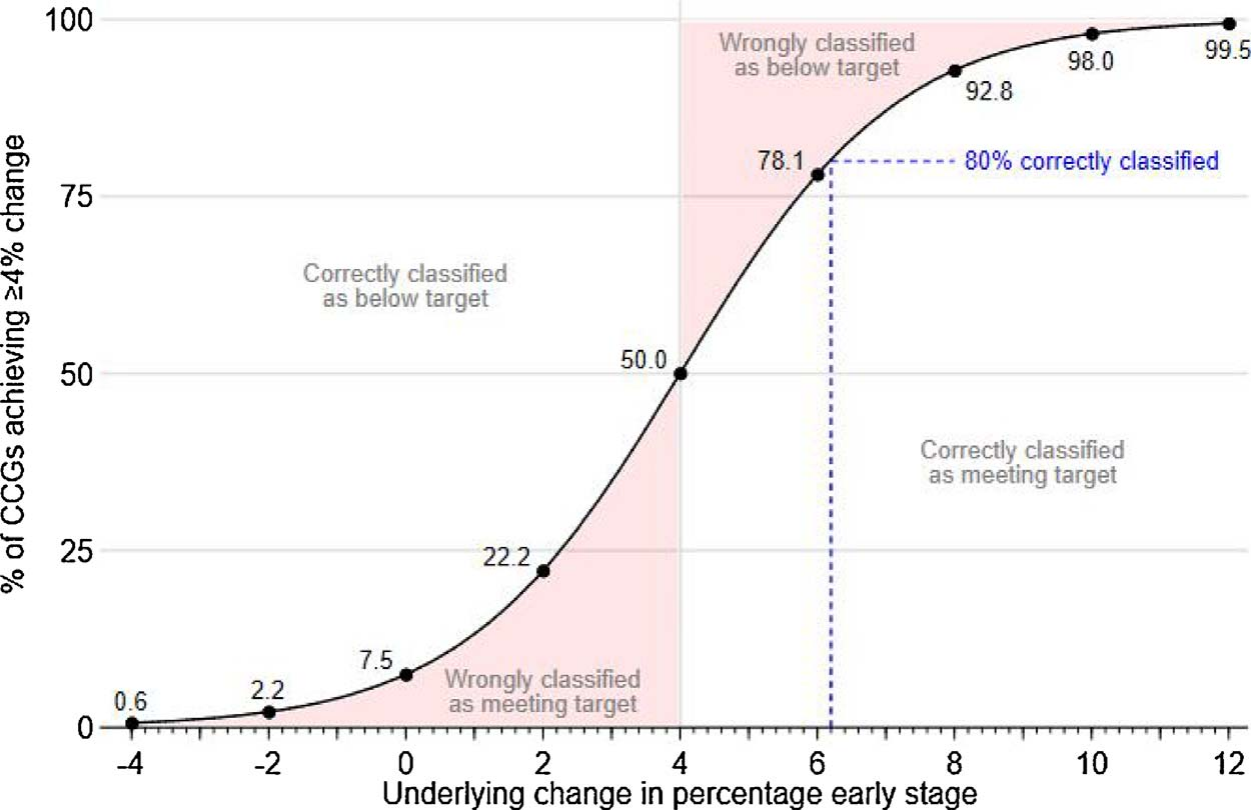
Expected percentage of CCGs with observed increases in the early stage percentage of 4 percentage points or more, given uniform national changes of between −4 and +12 percentage points. For example, for a typical CCG to have an 80% chance of being classified as achieving a 4%-point increase (blue dashed line), it would need to have an underlying increase of 6.2%-points. (For interpretation of the references to colour in this figure legend, the reader is referred to the web version of this article.)

For the 4% year-on-year increase criterion of the CCG Quality Premium, misclassification rates depend on the size of underlying changes in performance expected in the long-term for individual CCGs as well as CCG size. If the CCGs' underlying performance did not change, then with very large sample sizes we would not expect to see any CCGs meet this target. However, based on the actual sample sizes for one year of data we would expect 8% of CCGs to be misclassified as meeting the target if the underlying performance did not change for any CCG (Fig. 4). Furthermore, for a CCG to have an 80% chance of meeting the 4% improvement target they would have to improve their underlying performance such that they increased the percentage of cases diagnosed at early stage by 6.2% (Fig. 4).

## 4 Discussion

The current specification of the early stage indicator for English commissioning organisations is biased due to organisational variation in stage completeness. For the period we examine, the degree of bias is so large that it dominates the variability in this indicator. An alternative specification of the indicator based only on tumours with recorded stage is substantially less biased. Nonetheless, such complete-case indicators will not be reliable when based on one year of data, and will be associated with a high degree of random misclassification if used in pay-for-performance schemes. Complete-case indicators will be suitable for public reporting if based on three-year reporting periods. Timely early stage indicators suitable for pay-for-performance use are not feasible.

There are no previously published evaluations of the bias or reliability of indicators of cancer stage at diagnosis. Many studies have evaluated the reliability of other performance indicators in healthcare for physicians [7], [9], hospitals [23], [24], and general practices [8], [21] – including for several diagnostic activity indicators reported in the Cancer Services Public Health Profiles [19]. Bias due to missing data is also a common problem for measures based on routinely-collected data, and multiple imputation in particular is commonly used to correct this in cancer registry data [4], [25], [26].

The key strength of our study is that we use the same English cancer registry data as the early stage indicator, ensuring our results are directly relevant to the current public reporting and pay-for-performance schemes in England. The main weakness is the lack of an objective gold standard for assessing bias in the indicator. Our estimates of bias under different specifications of the indicator are based on comparisons with complete data produced using multiple imputation, as by definition we do not know the stage of tumours with no recorded stage. This approach could itself be biased if the ‘missing at random’ assumption does not hold, but this is mitigated by the inclusion of important auxiliary information in the imputation process [15], [16], [25].

As we had no data on successive years, we only estimated true misclassification rates against the 60% early stage target, but as we have shown, CCGs may be additionally misclassified when considering the 4% early stage improvement criterion. The degree of misclassification we report represents an under-estimate.

Among the 10 cancer sites included in the current indicators, some have higher than average proportion of late stage disease (e.g. lung cancer) whereas the opposite is true for other sites (e.g. breast cancer). The indicator does not take into account between-CCG variation in site-specific incidence or in patient demographics, and this may reduce the validity of the current indicator for comparing CCG performance [27], [28]. Adjusting for case-mix factors would be expected to reduce variation between organisations, and so a potential case-mix adjusted indicator might be more valid but less reliable. Future studies should establish the degree by which case-mix drives apparent organisational attainment and potential implications for public reporting conventions.

Continuing improvements in stage completeness in English cancer registry data will reduce the size and the variation of bias in the missing-is-late approach. However, bias due to missing stage information under this approach will remain a major problem until all CCGs have very similar stage completeness rates. In our study year the alternative complete-case approach has less bias than the current missing-is-late approach even for CCGs with very high stage completeness, and so would be expected to remain the best option as stage completeness continues to improve.

Aggregating 3 years of data will produce a reliable early stage indicator, suitable for use in public reporting, and we endorse this approach. Pay-for-performance schemes for Clinical Commissioning Groups should not use the early stage indicator, as sufficiently reliable indicators require more than eight aggregate years of data which greatly limits potential uses. The resulting high levels of misclassification on the indicator when based on a single year mean that many CCGs will receive financial rewards despite their underlying performance being below the pay-for-performance threshold. The opposite is also true, i.e. some CCGs should be rewarded but will not be.

Appropriate process indicators could give more accurate, reliable, and timely information about local diagnostic performance for cancer [29], [30], where there are clear links between processes and improved stage at diagnosis, survival, or quality of life. Screening coverage, for example, is a useful measure for breast, colorectal and cervical cancers [31], [32]. Other examples might include organisational measures of use of endoscopies or urgent referrals for suspected cancer (otherwise known as ‘two-week-wait’ referrals), as they are associated with clinical outcomes [33], [34]. More generally, there is a need for research to identify diagnostic process indicators which are truly linked to better outcomes for cancer patients, and to identify the organisations best-placed to improve local and national performance.

The development of indicators of cancer diagnosis must involve the evaluation and correction of issues of bias and low reliability. The methods we have highlighted here allow for investigation of these problems, and should form part of the process for the development of such indicators before their introduction into practice. Organisations should not be ranked on severely biased quality measures, and financial incentives should only be linked to highly reliable indicators. Cancer stage indicators should not form part of pay-for-performance schemes for CCGs, and public reporting of the early stage indicator should use three-year reporting periods and be calculated as the percentage of staged tumours diagnosed at an early stage.

## Authorship contribution statement

GL and GAA conceived the study. GAA and MB designed the study. MB and DG analysed data. All authors contributed to decisions about data analysis interpretation and drafted the article. All authors approved the final version for submission.

## Conflicts of interest

None.

## Figures and Tables

**Fig. A2:**
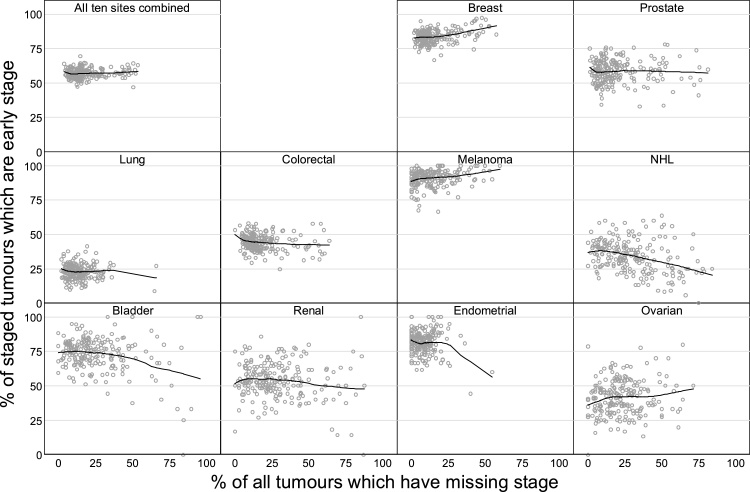
Percentage of staged tumours which were stage 1 or 2 against percentage of all tumours which were staged, by CCG, England 2013, with LOESS line.

**Table A1:** Studies evaluating bias introduced by missing data in cancer registry data.

First Author	Year published	Country	Setting	What was imputed	Summary
He Y	2008	US	Regional, California	Indicators of receiving chemotherapy or radiotherapy treatment (colorectal cancer), outcome variables	Correcting under-reporting using internal gold standard.
Krieger N	2008	US	Regional, California	ER-status (breast cancer), outcome variable	Records with missing ER status bias complete-case analysis
Nur U	2010	UK	1 English registry (NWCIS)	Stage (colorectal cancer), covariate	Complete-case analysis is likely to be biased. Indicator methods give spurious precision levels. MI allows inclusion of more information (leading to higher precision than complete-case). MAR assumption probably reasonable, but further research valuable.
Eisemann N	2011	Germany	1 German registry (Schleswig-Holstein)	Simulation (truly MAR), based on real data on breast cancer and melanoma. Stage was imputed by various methods (multinomial logistic; PMM; random forests) with various levels of missing data. Stage was used as outcome (incidence counts) and covariate (survival analysis)	MI is superior to simpler methods for handling missing data. MI using random forests does not perform well (and is associated with model convergence problems).
Howlader N	2012	US	13 SEER registries	ER-status (breast cancer), outcome variable but also for re-use by other researchers	Demonstration with incidence trends. Include the cancer registry in imputation when data from more than one registry are imputed.
Falcaro M	2015	UK	4 English registries	Simulation, based on real data. Stage was imputed by various methods under various (MAR) missingness mechanisms.	Ordinal logistic model is inadequate. Multinomial logistic model works well. Use of Nelson-Aalen estimate of cumulative hazard is recommended.
Andridge R	2016	US	13 SEER registries	ER-status (breast cancer), as outcome variable, using PMM under MAR and various MNAR assumptions	In SEER 1992–2012 breast cancer data, MAR and MNAR approaches give broadly similar results.
Falcaro M	2017	UK	4 English registries	Simulation, based on real data. Stage was imputed by various methods under various (MAR) missingness mechanisms, and the bias in different approaches to imputation was compared.	Can use imputation with non-congenial analysis methods (in this case, Pohar-Perme net survival estimation) to avoid bias associated with “missing indicator” approaches.

ER: Estrogen Receptor.

MAR: Missing At Random.

MNAR: Missing Not At Random.

PMM: Predictive Mean Matching.

**Table C1:** Number of organisations, staged tumours per organisation, odds ratios over estimated underlying distribution of organisational performance, quartiles of the reliability of the complete-case early stage indicator, and the number of tumours and associated aggregated years of data for 50%, 70%, 90% and 100% of organisations to have reliability of 0.7 or higher or of 0.9 or higher, for CCGs, local authorities and general practices.

		CCG	LA	GP
Units with staged tumours		209	326	8075
Staged tumours per unit	Minimum	125	12	1
	25th percentile	479	311	9
	Median	691	427	17
	75th percentile	943	634	30
	Maximum	3575	2992	150
Odds ratio over unit distribution*	75th/25th percentiles	1.16	1.18	1.29
	95th/5th percentiles	1.43	1.49	1.85
Reliability	Minimum	0.26	0.04	0.01
	25th percentile	0.58	0.53	0.06
	Median	0.66	0.61	0.12
	75th percentile	0.73	0.70	0.20
	Maximum	0.91	0.92	0.56
Tumours required for reliability 0.7	50% of units	803	641	280
	70% of units	812	652	302
	90% of units	833	668	358
	All units	926	784	1546
Data years required for reliability 0.7	50% of units	1.2	1.5	17.1
	70% of units	1.5	2.0	30.2
	90% of units	2.3	2.7	89.5
	All units	6.6	53.7	269.0
Tumours required for reliability 0.9	50% of units	3095	2470	1078
	70% of units	3132	2514	1165
	90% of units	3210	2575	1380
	All units	3570	3022	5963
Data years required for reliability 0.9	50% of units	4.5	5.8	65.8
	70% of units	5.6	7.5	116.4
	90% of units	8.7	10.5	345.0
	All units	25.3	206.8	1,035.0

* *p <* 0.0001 across CCGs, LAs and GPs.

**Table C2:** National number of diagnoses and median reliability of complete-case composite and site-specific early stage indicators for general practices, CCGs and local authorities, with number of years of data at current completeness levels required for reliable indicators for 70% of organisations.

Cancer site	Tumours			Median reliability			Years of data required for reliable indicators (*λ* ≥ 0.7) for 70% of organisations		
	Total	Staged	Stage 1–2	GP	CCG	LA	GP	CCG	LA
All ten sites combined	208,141	172,001	98,780	0.12	0.66	0.61	30.2	1.5	2.0
Breast	44,558	37,465	31,635	0.08	0.59	0.42	28.7	2.3	4.8
Prostate	39,934	32,859	19,422	0.05	0.71	0.62	75.3	1.3	1.9
Lung	35,972	31,234	7307	0.02	0.44	0.32	142.0	4.0	7.0
Colorectal	33,477	27,719	12,398	0.04	0.26	0.15	92.0	8.7	17.3
Melanoma	12,245	10,520	9591	0.10	0.26	0.19	34.0	9.7	19.2
NHL	11,222	8080	2916		0.33	0.24		7.0	10.8
Endometrial	7232	6615	5405		0.10	0.06		30.6	50.1
Bladder	8669	6505	4835		0.25	0.14		10.3	22.7
Renal	8368	5970	3202		0.28	0.21		8.1	13.4
Ovarian	6464	5034	2069		0.14	0.14		20.7	21.6

**Table C3:** Estimated number of true positives, false positives, true negatives and false negatives, with associated sensitivity, specificity, positive predictive value and negative predictive values (95% confidence intervals), for the 60% early stage target given performance similar to 2013 and tumours counts as in 2013 for reporting periods of 1, 2.5 and 9 years.

Reporting period	1 year		2.5 years		9 years	
	Expected value	(95% CI)	Expected value	(95% CI)	Expected value	(95% CI)
True positives	21	(13, 50)	23	(15, 32)	25	(16, 35)
False positives	19	(11, 28)	11	(6, 18)	5	(1, 10)
True negatives	161	(149, 172)	169	(157, 179)	175	(164, 185)
False negatives	8	(3, 14)	6	(2, 11)	4	(1, 8)
Sensitivity	0.73	(0.56, 0.89)	0.80	(0.64, 0.93)	0.88	(0.73, 0.97)
Specificity	0.89	(0.85, 0.94)	0.94	(0.90, 0.97)	0.97	(0.94, 0.99)
Positive predictive value	0.52	(0.37, 0.68)	0.67	(0.50, 0.82)	0.83	(0.68, 0.95)
Negative predictive value	0.95	(0.92, 0.98)	0.97	(0.94, 0.99)	0.68	(0.96, 0.99)

**Figure:**
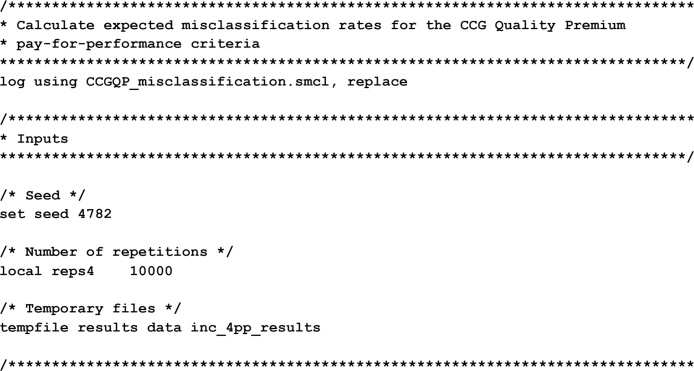


**Figure:**
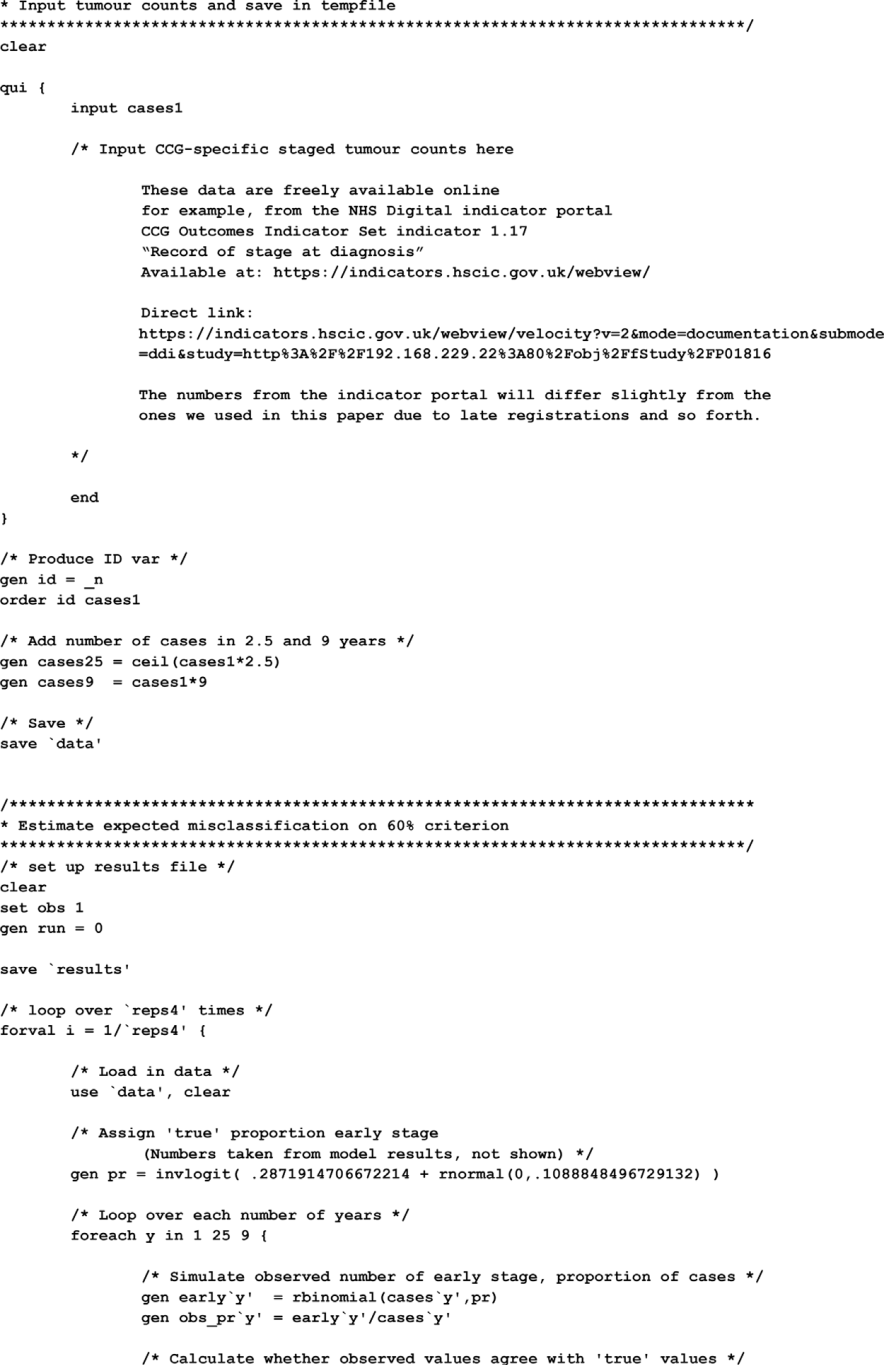


**Figure:**
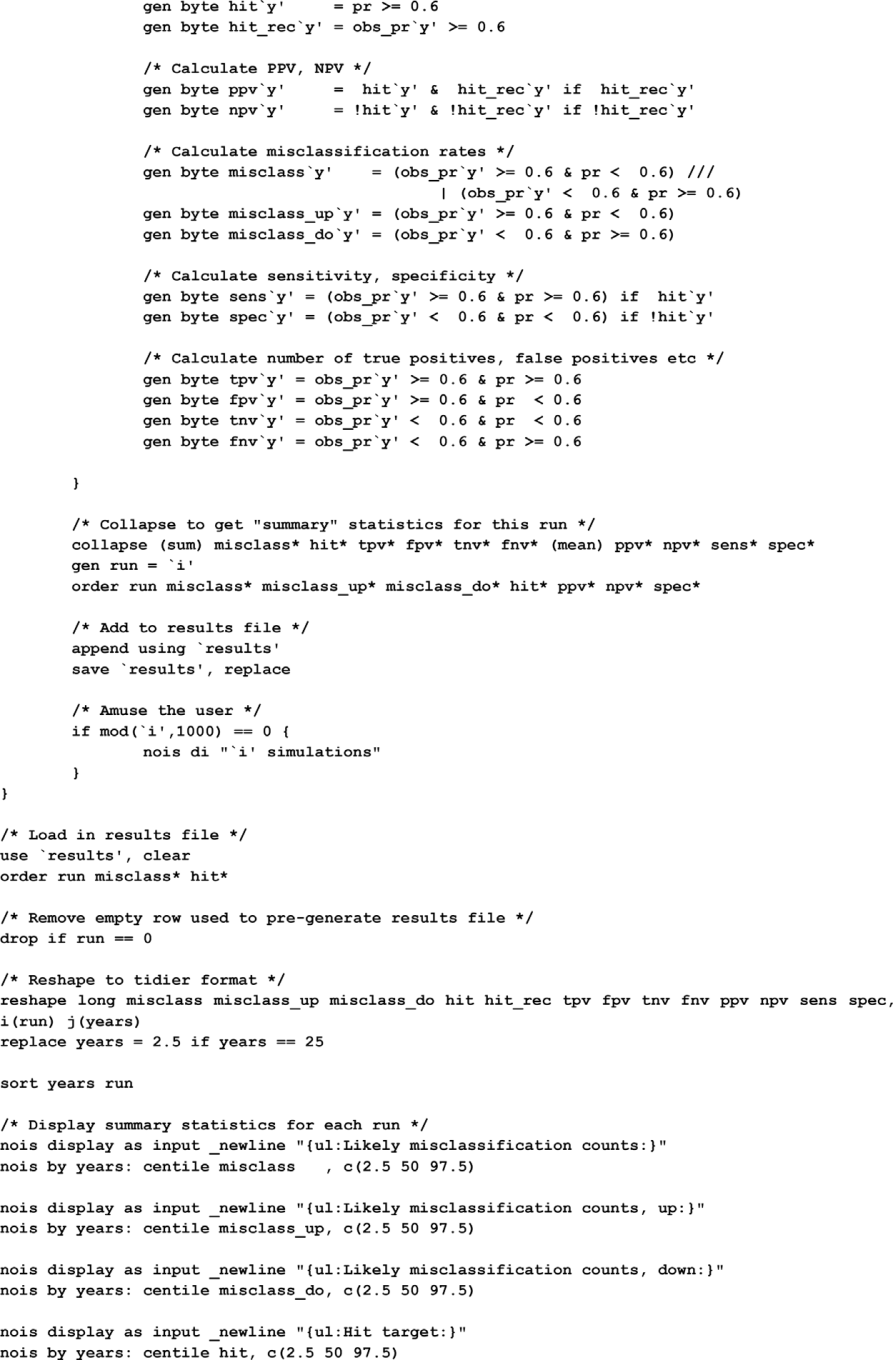


**Figure:**
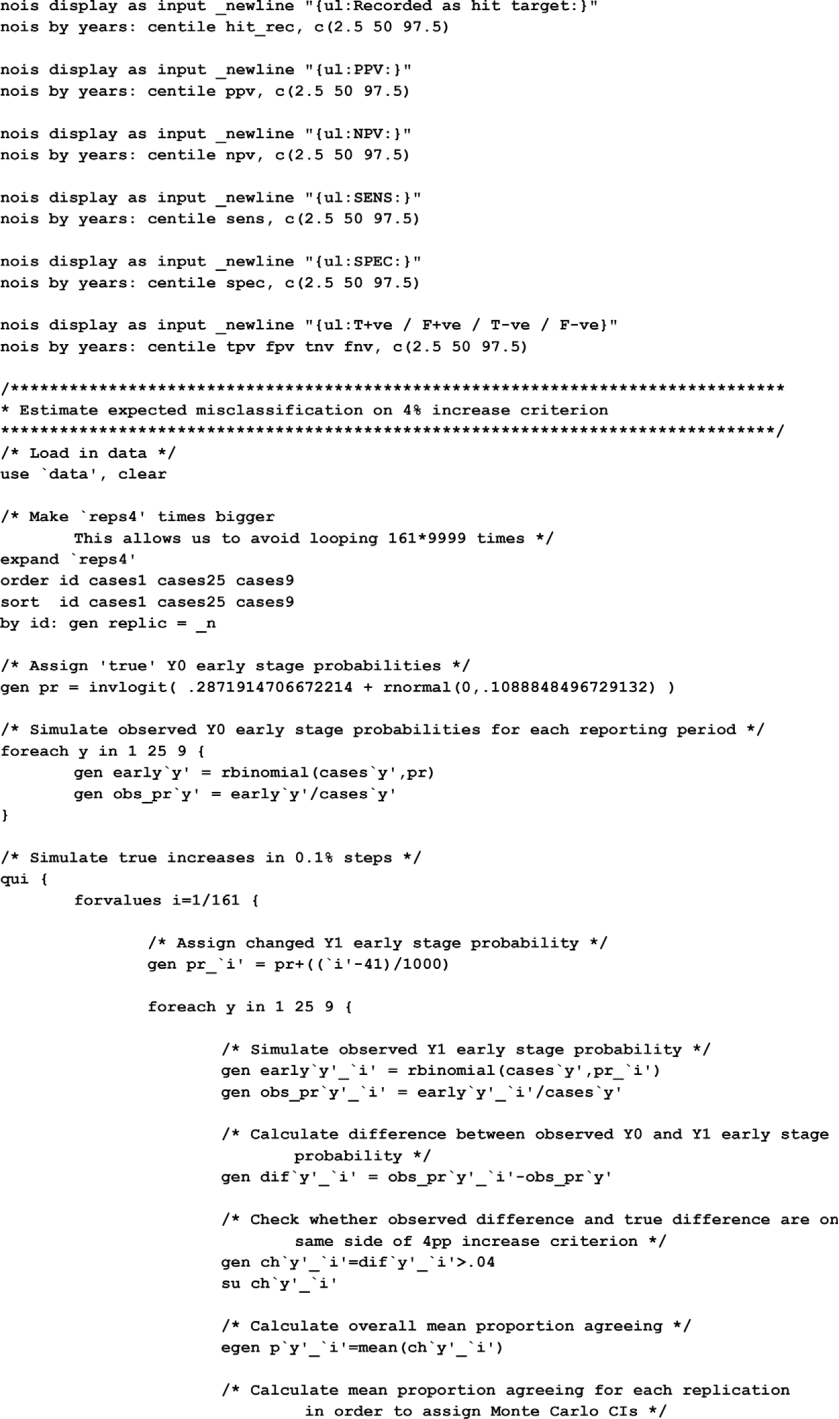


**Figure:**
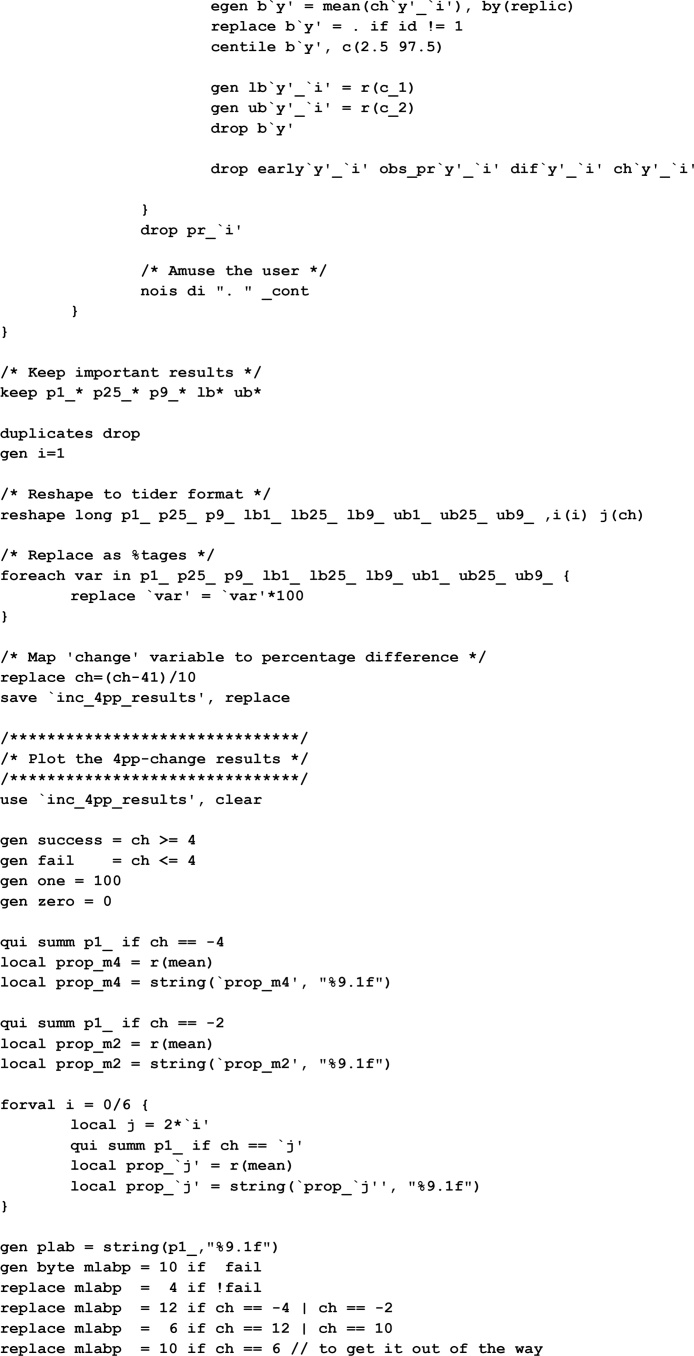


**Figure:**
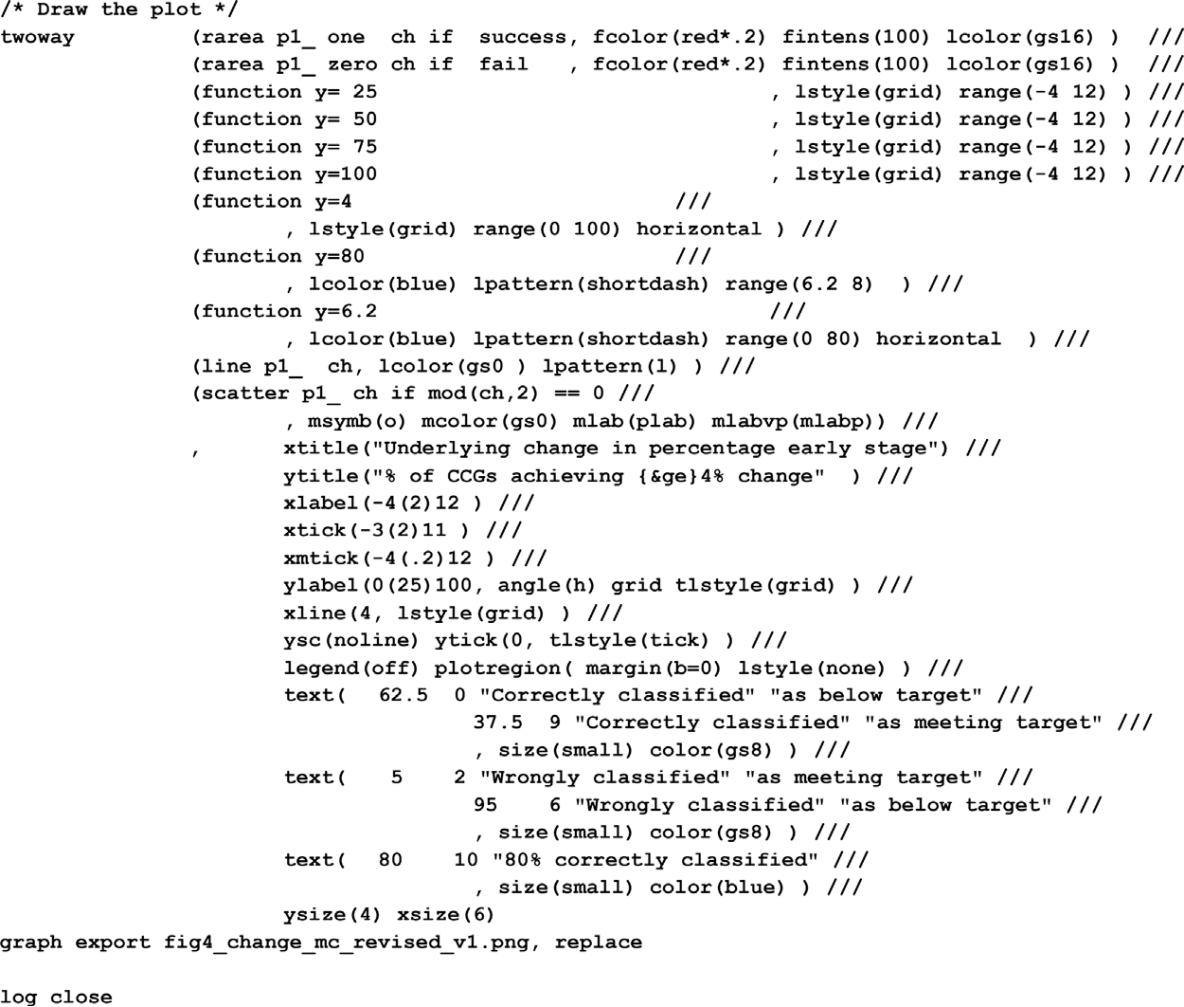


## Appendix A Details of multiple imputation of stage for patients with tumours with no recorded stage information

Stage data were 82% complete overall, with at least 70% completeness for each cancer site. However, stage completeness and the distribution of stage at diagnosis where known varied substantially by site (Fig. A1), and stage completeness also varied substantially by CCG (Fig. A2).

Multiple imputation is a recommended method for handling missing stage information in cancer registry data (Table A1). We created a binary stage variable being ‘early’ (TNM stages 1 or 2) or ‘late’ (TNM stages 3 or 4) stage. Imputation was performed separately for each cancer site, splitting colorectal cancer into colon and rectal cancer.

We used logistic regression to impute the binary indicator of early stage at diagnosis on:

• CCG of patient at diagnosis
• Region of residence of patient at diagnosis
• Sex of patient
• Interaction between sex and region
• Age group of patient at diagnosis (30–39, then five-year age groups, then 90–99, except for prostate and bladder cancer where the youngest age group was 30–44 due to smaller numbers in this age range)
• Interaction between age group and region
• Deprivation group, fifth of the income domain of IMD 2010
• Interaction between deprivation group and region
• Ethnicity of patient (white or non-white)
• Interaction between ethnicity and region
• Nelson-Aalen estimate of cumulative hazard, censored at 365 days after diagnosis
• Indicator of death within 365 days after diagnosis
• Indicator of death within 30 days after diagnosis (not included in imputation of stage for endometrial cancer or melanoma)
• Basis of diagnosis (non-microscopic/microscopic, not included in imputation of stage for endometrial cancer or melanoma)
• Screening detection status (for breast, colon and rectal cancer only)
• Tumour grade (1/2/3/4, not considered for melanoma)

We only included patients aged 30–99 at diagnosis. We felt that predictors of stage at diagnosis for patients outside this age range may not reflect those of more typical patients. There were few patients either aged 29 and under (1591 of 208,141, 0.8%) or 100 and older (104 of 208,141, 0.05%), so separate imputation was not feasible.

Screening detection status was applicable for breast, colon and rectal cancers. For melanoma and endometrial cancer, early mortality and non-microscopic diagnosis were both extremely rare and the inclusion of such indicators led to problems with model convergence. For melanoma, tumour grade is both less clinically relevant and had low completeness..

All variables used in imputation models were complete, except for tumour grade. For cancer sites other than melanoma, we used predictive mean matching to impute tumour grade based on the (possibly imputed) binary indicator of early stage at diagnosis and on the other variables and interactions used in imputing stage.

Thus for melanoma we used multiple imputation by logistic regression, while for other sites we used multiple imputation by chained equations. We used ten iterations of the chain as burn-in, having previously checked graphically that doing so led to convergence.

## Appendix B Organisation-level reliability for binary indicators

The statistical reliability of a measure generally indicates its reproducibility (consistency) in repeated measurement and its robustness to random measurement error. Here we are concerned with organisation-level reliability, also termed unit-level reliability where units could be commissioners, providers, or geographical areas. In the context of our study, organisation-level (or Spearman-Brown) reliability represents the extent to which measured percentages of cancer patients diagnosed in early stage reflect true differences between organisations, as opposed to random (i.e. chance) variation. Alternatively, the Spearman-Brown reliability is the proportion of the observed organisational variation not due to chance.

Poor reliability often arises when the typical number of cases per organisation (in a given reporting period) is small. The problem is further exacerbated when small sample sizes are combined with limited variation between organisations. Reliable indicators can help to classify organisational performance and thus enable accurate targeting of improvement efforts and rewards. Conversely, using unreliable indicators can lead to harm through wasting of scarce improvement resources and related opportunity costs. Further, misclassified ‘poorly performing’ organisations may sustain unfair reputational or financial loss [6], [9].

Reliability takes a value between 0 and 1, with higher values denoting more reliable indicators. A reliability of 0.5 indicates that half of the observed variance is due to chance. A reliability of 0.7 is often required for public reporting of indicators, while a reliability of 0.9 may be required for pay-for-performance use [6], [20], [21]. Organisation-level reliability *λ*_*i*_ for organisation *i* is defined as

$$\lambda_{i}=\frac{\text{between-organisation variance}}{\text{between-organisation variance+}\frac{\text{within-organisation variance}}{n_{i}}}$$

where *n_i_* = achieved sample size for organisation *i*.

For continuous indicators, this calculation is straightforward [6]. For binary indicators, the within-organisation variance will depend directly on the level of achievement at each individual organisation, according to the binomial distribution [18], [20]. It is important to note that as reliability depends on both the organisational sample size and organisational achievement it is specific to each organisation rather than to the indicator as a whole.

We used mixed effects logistic regression models to estimate the organisation-level variance on the log-odds scale ($\hat{\sigma^{2}}$). Reliability is then given by

$$\lambda_{i}=\frac{\hat{\sigma^{2}}}{\hat{\sigma^{2}}+\frac{1}{\hat{\pi_{i}}\times \left(1-\hat{\pi_{i}}\right)\times n_{i}}}$$

where $\hat{\pi_{i}}$ is the observed performance of organisation *i* on the indicator as a proportion [18]. From this formula it can be seen that higher reliability can be achieved by increasing the between-unit variation or by increasing sample sizes. Additionally, for binary indicators, higher reliability is achieved with performance closer to 50%.
